# Leishmaniasis and Chagas disease: Is there hope in nanotechnology to fight neglected tropical diseases?

**DOI:** 10.3389/fcimb.2022.1000972

**Published:** 2022-09-16

**Authors:** Debora B. Scariot, Austeja Staneviciute, Jennifer Zhu, Xiaomo Li, Evan A. Scott, David M. Engman

**Affiliations:** ^1^ Department of Biomedical Engineering, Chemistry of Life Processes Institute, and Simpson Querrey Institute, Northwestern University, Evanston and Chicago, IL, United States; ^2^ Department of Pathology and Laboratory Medicine, Cedars-Sinai Medical Center, Los Angeles, CA, United States; ^3^ Department of Pathology, Northwestern University, Chicago, IL, United States

**Keywords:** Chagas disease, leishmaniasis, nanotechnology, drug delivery systems, trypanosomatids

## Abstract

Nanotechnology is revolutionizing many sectors of science, from food preservation to healthcare to energy applications. Since 1995, when the first nanomedicines started being commercialized, drug developers have relied on nanotechnology to improve the pharmacokinetic properties of bioactive molecules. The development of advanced nanomaterials has greatly enhanced drug discovery through improved pharmacotherapeutic effects and reduction of toxicity and side effects. Therefore, highly toxic treatments such as cancer chemotherapy, have benefited from nanotechnology. Considering the toxicity of the few therapeutic options to treat neglected tropical diseases, such as leishmaniasis and Chagas disease, nanotechnology has also been explored as a potential innovation to treat these diseases. However, despite the significant research progress over the years, the benefits of nanotechnology for both diseases are still limited to preliminary animal studies, raising the question about the clinical utility of nanomedicines in this field. From this perspective, this review aims to discuss recent nanotechnological developments, the advantages of nanoformulations over current leishmanicidal and trypanocidal drugs, limitations of nano-based drugs, and research gaps that still must be filled to make these novel drug delivery systems a reality for leishmaniasis and Chagas disease treatment.

## Introduction

Neglected tropical diseases (NTDs) comprise a group of 20 diseases that affect 1.7 billion people living in middle- and low-income tropical countries. It is estimated that everyone living in extreme poverty (income < US $1.90/day) have one or more NTD (Hotez et al., 2020). Leishmaniasis and Chagas disease (CD) are NTDs caused by the trypanosomatid protozoa parasites *Leishmania* spp. and *Trypanosoma cruzi*, respectively. Despite numerous efforts by global agencies to eradicate these pathogens, the incidence of cutaneous leishmaniasis and the prevalence of CD increased between 2006 and 2016 according to the Global Burden of Diseases report (Vos et al., 2017; Lin et al., 2022). The rising morbidity rates indicate how poverty may weaken entire populations by limiting access to healthcare, housing, sanitary conditions, and education, perpetuating a poverty cycle by limiting productivity and impairing physical and cognitive development (Souto et al., 2019; Ahmed et al., 2022).

The status of leishmaniasis and CD as NTDs emphasizes the need for affordable, safe, and effective treatments for these diseases since current treatments are inadequate or inaccessible. The toxicity of current therapies contributes to significant side effects, which often necessitate treatment termination. Decades of continuous use of the same drugs has led to the development of drug-resistant strains, further justifying the need for new therapies (Field et al., 2017; Santos et al., 2020b). Additional challenges for leishmaniasis and CD treatment include patient compliance with therapy, drug instability, complex or unknown pathogenic mechanisms, and environmental factors that impact transmission of infection (Field et al., 2017; Menna-Barreto, 2019; Souto et al., 2019). Nevertheless, the association of these diseases with poverty represents a major obstacle to the research and development of drugs, vaccines, and diagnostic methods to treat and prevent them (Weng et al., 2018; Winkler, 2021).

As drug discovery and development is a long and expensive process, nanotechnology has emerged as a promising approach to overcoming limitations associated with the current drug formulations, without the need to create new drugs from scratch. The flexibility inherent in nanotechnology platforms permits tailoring of the novel nanoformulation with respect to the material (lipid-based polymeric, inorganic), charge, size, and surface modifications, giving rise to high-precision therapies and repurposing of therapeutics (Scott et al., 2017; Burke et al., 2022; Vincent et al., 2022). The encapsulation of drugs within these systems helps to increase cargo stability and improve pharmacokinetic aspects, such as prolonging circulation time and modifying biodistribution (Mitchell et al., 2021). Nanotechnology has the potential to reduce toxic effects and potentialize the drug efficacy by delivering low drug doses to target-infected cells/tissues (Barry and Sadler, 2013).

Since 1995, when the first nanomedicine, Doxil, was commercialized, approximately 60 nanomedicines and 100 nanomedicine-related products have been registered around the world (Abdellatif and Alsowinea, 2021; Shan et al., 2022). The most recent approvals were given to mRNA-Covid-19 vaccines that use lipid nanoparticles to deliver mRNA to monocytes and dendritic cell subsets for immunization against SARS-CoV-2 (Shan et al., 2022; Trougakos et al., 2022). Yet after almost two decades since the approval of Doxil, no nanomedicine has been approved for the treatment of CD and only the nanomedicine liposomal amphotericin B (LAmpB)has been approved for the treatment of leishmaniasis, despite the very high cost of this treatment (Prasanna et al., 2021; Assolini et al., 2022).

In this sense, is there hope in nanotechnology to fight neglected tropical diseases? Does nanotechnology have potential to fill gaps in current pharmacotherapy to treat leishmaniasis and CD? To answer these questions, we will discuss the most publicized nanomedicines and the most recent advances in nano-based drug delivery systems to treat CD and leishmaniasis in this review. Clinical trials and animal studies will be reviewed and the specific advantages of nanotechnology will be highlighted in terms of remodeling drug biodistribution and pharmacokinetics to improve drug efficacy, reduce toxicity, and modulate the inflammatory response. Main aspects and findings of all studies discussed here are summarized in **Table 1**.

**Table 1:** Overview of the reviewed sources.

Disease	Particulate System	Encapsulated Cargo/Administration route	Activity/Application	Results	Ref
**Leishmaniasis**	Liposomes	Amphotericin B (iv)	Parasiticidal (drug delivery) Post-Kala-Azar Dermal leishmaniasis	•the combination of AmpB-loaded liposomes and Miltefosine cured 100% of infected humans •reduced time of treatment prevented relapses •no signals of toxicity	(Ramesh et al., 2020)
	Liposomes	Amphotericin B (iv)	Parasiticidal (drug delivery)Visceral leishmaniasis*(L. donovani)*	•acute stage: up to 90% of splenic and hepatic parasitemia suppression •chronic stage: up to 62% hepatic parasite burden reduction; lower AmpB concentrations in plasma, liver, and spleen •hepatic and splenic inflammation prevented the AmpB accumulation in the liver and spleen	(Voak et al., 2017)
	Hyaluronic acid-coated liposomes	Quinoxaline derivative (topical)	Parasiticidal (drug delivery)Cutaneousleishmaniasis*(L. amazonensis)*	•liposome accumulation in liver, spleen, and infected lesion •limited skin permeation	(de Oliveira et al., 2020)
	Chitosan nanoparticles	Amphotericin B (iv and topical)	Parasiticidal (drug delivery) Cutaneous leishmaniasis (*L. major*)	•slow drug release in a pH-sensitive manner •up to 99% of lesion size and parasite load reduction •similar antileishmanial activity to AmBisome •limited skin permeation	(Riezk et al., 2020)
	Chitosan nanoparticles	S-nitrosothiol (topical)	Parasiticidal (immunomodulation) Cutaneous leishmaniasis (*L. amazonensis*)	•sustained NO release •parasite load reduction of ~49% in the first 5 days after one topical application	(Cabral et al., 2021)
	Copper nanoparticles	Copper (topical)	Parasiticidal (oxidative stress) Cutaneous leishmaniasis (*L. major*)	•CuNPs combined with intralesional meglumine antimoniate promoted 100% recovery of infected mice •the parasiticidal mechanism is based on the Cu ability to trigger the production of NO	(Albalawi et al., 2021)
	Mannosylated thiolated chitosan nanoparticles	Meglumine antimoniate (p.o.)	Parasiticidal (drug delivery)Visceral leishmaniasis(*L. donovani*)	•increased intestinal permeation and drug bioavailability •targeted macrophages •suppressed the parasite burden in spleen and liver compared to the standard treatment	(Shoaib Sarwar et al., 2020)
	Liposomes	Fullerol (ip)	Parasiticidal and inflammation control (immunomodulation) Acute visceral leishmaniasis (*L. amazonensis*)	•elimination of hepatic parasites in 100% of animals suppressed splenic infection •increased Th-1 and Th-2 response	(Ramos et al., 2021)
	Yeast cell wall particles	Thiophene (p.o.)	Parasiticidal (drug delivery and tissue targeting) Immunomodulation Visceral leishmaniasis *(L. infantum)*	•reduction of splenic and hepatic parasite burden •increased of Th-1 response	(Scariot et al., 2019b)
	Lipid nanoparticles	Diselenide (p.o.)	Parasiticidal (drug delivery) Visceral leishmaniasis *(L. infantum)*	•efficient oral drug delivery: enhanced intestinal permeability and bioavailability •95% of parasite burden reduction after 5 doses	(Etxebeste-Mitxeltorena et al., 2021)
	Carboxymethyl chitosan liposomes	Amphotericin B (p.o.)	Parasiticidal (drug delivery) Visceral leishmaniasis *(L. donovani)*	•93.5% of hepatic parasite burden reduction with no toxicity •sustained drug release, elevated stability and bioavailability.	(Singh et al., 2022)
	Carboxymethyl cellulose/polyvinylpyrrolidone microneedles	Amphotericin B (transdermal)	Skin penetration (drug delivery) Cutaneous leishmaniasis *(L. major)*	•sustained drug release through the skin •microneedles were able to deliver the drug in the epidermis and dermis layers	(Zare et al., 2021)
	Solid lipid nanoparticles modified with β-cyclodextrin	Melatonin plus Amphotericin B (p.o.)	Parasiticidal (drug delivery) Visceral leishmaniasis *(L. donovani)*	•inhibition of hepatic parasitic burden •parasiticidal effect was potentialized by melatonin	(Parvez et al., 2021)
	Lipid nanocarriers	Ursolic acid	Parasiticidal and Immunomodulation (drug delivery) Visceral leishmaniasis *(L. infantum)*	•no toxicity and controlled inflammatory response •reduction of splenic and hepatic parasitism	(Jesus et al., 2021)
	Maghemite/polyethylenebyimine nanoparticles (Nano-Leish-IL)	No drug (topical)	Parasiticidal Cutaneous leishmaniasis *(L. major)*	•cutaneous lesion volumes and the parasitic burden were reduced by Nano-Leish-IL treatment •polyethylenimine promoted a cytolytic effect on the parasites	(Kannan et al., 2021)
	Poly-l-lactide -nanocapsules	Meglumine Antimoniate (ip)	Parasiticidal (drug delivery) Visceral leishmaniasis *(L. infantum)*	•reduction of parasite number in liver, spleen, and kidneys especially after 45 days of treatment •lower renal accumulation of meglumine antimoniate and a significant increase of its plasmatic half-life	(Cosco et al., 2021)
**Chagas Disease**	Hydrogel nano-porous particles Chunap	No drug	Diagnosis	•Chunap was able to concentrate *T. cruzi* antigens from urine of infected infants showing higher sensitivity than PCR •Chunap detected *T. cruzi* antigens in the urine of *T*. *cruzi*/HIV co-infected patients	(Castro-Sesquen et al., 2014; Castro-Sesquen et al., 2016)
	Indium Phosphide (InP) nanowires	No drug	Diagnosis of chronic CD	•detection of low levels of anti-*T. cruzi* antibodies in non-purified serum	(Janissen et al., 2017)
	Gold nanoparticles conjugated to silsesquioxanes	No drug	Diagnosis	•successful detection of anti-*T. cruzi* antibodies in the serum •hemocompatible and not toxic	(Lima et al., 2022)
	PEG-b-PPS polymersomes	Benznidazole (iv)	Parasiticidal (drug delivery) Acute CD (Y strain)	•loaded polymersomes were as effective as free BNZ using a dosage 466-fold lower than daily free BNZ •suppressed cardiac inflammation, heart and blood parasite burden •no signals of toxicity	(Li et al., 2021)
	Self-nanoemulsifying system	Ravuconazole (p.o.)	Parasiticidal (drug delivery) Acute CD (Y strain/Colombiana strain)	•increased dissolution rate of ravuconazole •increased cure rates after short-term treatment (30 days) •clearance of parasites after long-term treatment (40 days) •no signals of toxicity	(Spósito et al., 2017; Spósito et al., 2021)
	Nanoarchaeosomes	Imiquimod (sc)	Immunotherapy (drug delivery) Acute CD (RA strain)	•100% survival •promoted a protective T-helper response •reduction of inflammation/pro-inflammatory cytokines and fibrotic lesions in the cardiac and skeletal muscles	(Parra et al., 2020)
	PLGA-nanoparticles	Curcumin (p.o.)	Parasiticidal and Anti-inflammatory (drug delivery) Chronic CD (Brazil strain)	•the combination of curcumin-NPs and free benznidazole avoided heart injuries •downmodulation of cardiac inflammation and fibrosis	(Hernandez et al., 2021)
	Poloxamer (P-188) nanoparticles	Benznidazole (p.o.)	Parasiticidal (drug delivery) Acute CD (Nicaragua strain)	•lower levels of anti-*T. cruzi* antibody than free BNZ treatment •reduction in the Chagas disease reactivation after immunosuppression •decrease of heart inflammation and damage	(Rial et al., 2017)
	Poloxamer (P-188) nanoparticles	Benznidazole (p.o.)	Parasiticidal (drug delivery) Chronic CD (Nicaragua strain)	•elimination of parasitemia and Chagas reactivation •intermittent administration is more efficient than continuous administration •lower levels of IFN-γ and heart fibrosis	(Rial et al., 2020)
	Eudragit microparticles	Benznidazole (p.o.)	Parasiticidal and Antiinflammatory (drug delivery) Acute CD (Nicaragua strain)	•reduction in the parasite burden and anti-*T. cruzi* antibodies level •no heart damages •treatment prevented the progression to the chronic stage	(Rial et al., 2021)
	Multiparticulate polymeric system: Eudragit EPO-Eudragit L100	Benznidazole (p.o.)	Parasiticidal (drug delivery) Chronic CD (Tulahuen strain)	•higher efficacy than free benznidazole against cardiac parasites •lower cardiac and hepatic damage than free benznidazole •controlled release of BNZ and lower host oxidative stress	(Garcia et al., 2021)
	PLA-PEG nanocapsules	Lychnopholide (sesquiterpene lactone) (p.o.)	Parasiticidal (drug delivery) Acute and chronic CD (VL-10 strain)	•acute stage: suppressed blood parasitemia and cure rate of up 75%; reduction of cardiac inflammation and fibrosis •chronic stage: cure rate of up 87.5%; reduction of heart fibrosis and absence of heart inflammation	(Branquinho et al., 2020)

p.o., “per os”/by mouth; iv: intravenous; sc: subcutaneous; ip, intraperitoneal.

## Leishmaniasis

Leishmaniasis is a zoonotic infectious disease caused by more than 20 species of *Leishmania* and transmitted by over 90 species of phlebotomine sandflies, especially in low-income tropical countries. The World Health Organization estimates that 700,000 to 1 million new cases of leishmaniasis occur annually around the world, resulting in up to 30,000 deaths (WHO, 2022). Leishmania infection has three major clinical forms: cutaneous leishmaniasis (CL - localized and disseminated or diffuse), mucocutaneous leishmaniasis (MCL), and visceral leishmaniasis (VL). The development of a specific clinical manifestation depends on the Leishmania species involved, which induces a specific host immune response that also contributes to pathogenesis. Immunocompromised people, such as those with HIV infection, are more susceptible to developing a diffuse infection*. L. infantum* and *L. donovani* are species that cause VL, which can be fatal if not treated. The main symptoms of VL are hepatosplenomegaly, anemia and bleeding, and weight loss (Forrester et al., 2022). *L. mexicana* and *L. braziliensis* are likely to cause CL and MCL, respectively. CL and MCL cause skin and mucosal ulcers associated with an intense Th-1 inflammatory response that destroys the skin tissue and throat mucosa (Figueiredo et al., 2020; Kato et al., 2021). Disability, disfiguring scars causing social stigma and psychological consequences contribute to the current economic burden and overall impact of leishmaniasis (Okwor and Uzonna, 2016). Controlling the transmission cycle of Leishmania is challenging since many sylvatic animals, such as rodents, marsupials and bats, are natural reservoirs of this parasite (Roque and Jansen, 2014). Additionally, climate change has contributed to spreading the insect vector to extend the endemic area of leishmaniasis. Geographic areas experiencing war and terrorism are likely to develop leishmaniasis outbreaks as a result of social and healthcare system collapse promoted by these conflicts (Al-Salem et al., 2016; Al-Bajalan et al., 2018). No vaccine exists for human leishmaniasis, although there are three vaccines available to prevent leishmaniasis in dogs, the primary domestic Leishmania reservoir (Velez and Gállego, 2020).

**Figure 1:**
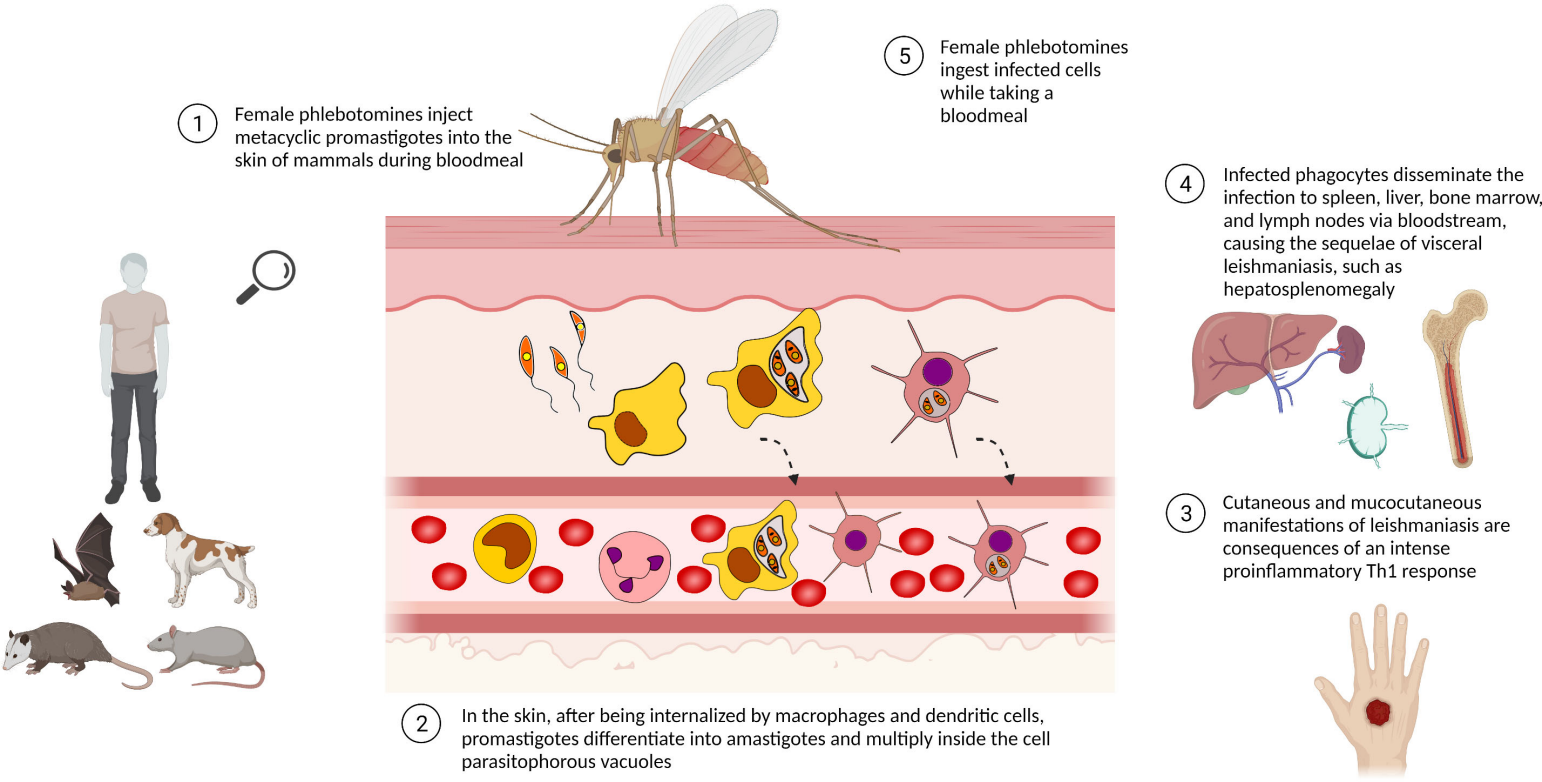
Life cycle of *Leishmania* spp. The life cycle of *Leishmania* spp. alternates between phlebotomine sandfly and mammalian hosts, e.g. sylvatic/domestic animals and human beings. Leishmania promastigotes are phagocytosed by mononuclear phagocytic cells, differentiating into amastigotes and multiplying by binary fission as an obligate intracellular parasite. The presence of a high number of intracellular parasites promotes the host cell disruption, releasing parasites able to infect other cells/tissues. Infected phagocytes can also reach lymphoid organs, disseminating the disease. Phlebotomine sandflies ingest Leishmania-infected cells while taking a bloodmeal and transmit leishmaniasis by biting man or other mammals. Created with Biorender.com.

Leishmania present a complex life cycle, alternating between invertebrate host –phlebotomine sandflies – and vertebrate mammal hosts, such as humans, sylvatic and domestic animals (**Figure 1**). Female sandflies inject elongated flagellated promastigotes of *Leishmania* into the mammalian host while they take a bloodmeal. After being phagocyted by mononuclear phagocytes, especially macrophages, promastigotes reside inside the phagosomes/parasitophorous vacuole. The mammalian host temperature and the acidic pH typical of parasitophorous vacuoles induce the promastigotes to differentiate into non-motile rounded amastigotes. These conditions also stimulate intense exocytosis of parasite signaling molecules, such as NF-ĸβ and gp63, to prevent the nitric oxide (NO) synthesis needed for typical macrophage parasiticidal activity. Therefore, by modulating the macrophage immunologic response, *Leishmania* survives inside host cells as an obligate intracellular parasite (Hassani et al., 2011; Burza et al., 2019).

Since the early 1990s, first-line drugs to treat leishmaniasis have been limited to two pentavalent antimonials – sodium stibogluconate and N-methyl glucamine antimoniate (Pund and Joshi, 2017). Both drugs are toxic and long-term treatment may be suspended to avoid life-threatening side effects, such as cardiac and hepatic dysfunction. The required parenteral administration impairs the treatment access given that healthcare infrastructure is unavailable in the most endemic remote areas. Originally developed as an antineoplasic, Miltefosine has been used as an alternative treatment for antimonial-resistant Leishmania strains in some countries, such as India, Nepal, and Bangladesh. The main advantage of Miltefosine over other leishmanicidal drugs is the convenient oral administration. Considered potentially teratogenic, subtherapeutic levels of Miltefosine resulting from the long elimination half-life leads to a selective pressure and emergence of Miltefosine-resistant Leishmania strains (Dorlo et al., 2012; Ponte-Sucre et al., 2017). As second-line treatments, amphotericin B (AmpB), pentamidine, paromomycin, and azoles show variable antileishmania efficacy, drug-resistant strains have been reported for all available leishmanicidal drugs. Drug combinations have been clinically applied to improve antileishmanial efficacy, as well as to delay the development of drug resistance and decrease side effects (Sundar and Chakravarty, 2015; Sundar and Singh, 2018).

In terms of nanotechnology, current leishmaniasis treatment relies on LAmpB/AmBisome formulations to reduce the side effects of AmpB deoxycholate such as nephrotoxicity and hematotoxicity. Specific types of leishmaniasis, such as post-visceral dermal kala-azar, may show limited susceptibility to LAmpB (Moulik et al., 2018). A clinical study revealed that a combination of LAmpB and miltefosine promoted regression of lesions and cure after 45 days of treatment, with no relapses or significant toxicity. On the contrary, 25% of patients treated with Miltefosine monotherapy for 90 days relapsed and developed mild gastrointestinal side effects (Ramesh et al., 2020). Measuring less than 100 nm, LAmpB is efficiently taken up by and accumulates within phagocytes from the liver and spleen. Liver and spleen inflammation is a classical symptom of chronic VL that can promote an erratic drug metabolism, biodistribution, and elimination. Voak et al. demonstrated that LAmpB is more effective if administered in the early stage of infection (before 21 days post-infection/dpi), since chronic leishmaniasis promotes physiological changes in the liver and spleen that interfere with LAmpB biodistribution. Plasma concentrations of AmpB, however, remain unaffected regardless whether the treatment is administered during the early or late stage of infection (Voak et al., 2017). These findings suggest the importance of evaluating biodistribution, pharmacokinetics, and pharmacodynamic parameters in Leishmania-infected animals, since drug and nanoparticle metabolism may be affected by the physiologic and metabolic disturbances caused by the infection itself.

Although LAmpB is readily available in developed countries, affordable drugs are sorely needed in low-income countries, where the tropical temperatures and lack of health care infrastructure require stable drug formulations for distribution, long-term storage and oral administration. Considering that drug bioavailability and efficacy depend on drug solubility, poorly soluble compounds have been discarded as potential drugs for oral administration (Sharma et al., 2016; De Rycker et al., 2018; Alqahtani et al., 2021).

Nanostructured lipid nanocarriers are more stable in gastrointestinal physiological conditions than liposomal systems, encouraging the investigation of their potential to deliver hydrophobic drugs by the oral route. As an example, selenocompounds showing low intestinal permeability and solubility in water show improved intestinal absorption, clearance, and bioavailability after oral administration when encapsulated in nanostructured lipid nanocarriers. Without causing any genotoxicity in healthy animals, five oral administrations of encapsulated selenocompound were sufficient to reduce the *L. infantum* burden in infected animals by up to 99.9%, which is similar to the effect of the standard Fungizone treatment after 10 i.v. administrations. Although there are no data regarding the accumulation of these nanocarriers in the primary organs targeted by visceral leishmaniasis (e.g. liver, spleen, and bone marrow), only selenocompunds that reached plasma selenium concentrations higher than the *in vitro* intramacrophage IC_50_ index showed significant antileishmania activity in infected animals (Etxebeste-Mitxeltorena et al., 2021). Singh et al. also developed a biocompatible and stable AmpB lipid-based nanocarrier for oral administration by grafting carboxymethyl chitosan (CMC) on the surface of lipid nanoparticles (CMC-AmpB-LV) (Singh et al., 2022). CMC grafting promoted a controlled release of AmpB and protected the nanovesicles against enzymatic degradation typically found in the gastrointestinal tract, with no significant drug release. CMC also increased the hydrophilicity of AmpB nanovesicles and prolonged their residence time on the intestinal mucosa by improving mucoadhesion, suggesting that CMC-AmpB-LV enhances the bioavailability of oral AmpB. *In vitro* assays confirmed that macrophages internalize the particles through clathrin-mediated endocytosis without damaging the cell membrane. The oral administration of CMC-AmpB-LV inhibited around 90% of the hepatic parasite burden of *L. donovani*-infected mice and no toxicity was detected, indicating that CMC nano-based formulations are a promising alternative for oral delivery of AmpB (Singh et al., 2022).

Solid lipid nanoparticles can also deliver more than one bioactive molecule simultaneously, which has considerable advantages for drug therapy. Melatonin and AmpB, an immunomodulator and a chemotherapeutic agent, respectively, were encapsulated into solid lipid nanoparticles surface-decorated with β-cyclodextrin (β-CD) and evaluated to treat murine visceral leishmaniasis through oral administration. A better bioavailability was expected from β-CD -decorated nanoparticles by enhancing water solubility and intestinal permeability. Data show that the Melatonin-AmpB association and the β-CD conjugation as well were crucial for the significant reduction in hepatic parasite burden. No evidence of renal and hepatic toxicity were observed (Parvez et al., 2021).

Natural molecules can also be encapsulated in lipid nanoparticles to improve water solubility and to reduce toxicity. Jesus et al. reported hepatic toxicity of the antileishmanial agent ursolic acid in a dose-dependent manner in golden hamsters. Toxicity was eliminated by encapsulation of ursolic acid in solid lipid nanoparticles. Additionally, ursolic acid-loaded nanoparticles measuring around 260 nm were able to reduce the hepatic and splenic parasite burden more efficiently than the standard AmpB treatment and free ursolic acid, promoting normalization of aspartate aminotransferase and creatinine levels normally elevated by infection. The efficient delivery of ursolic acid from lipid nanoparticles stimulated expression of IFN-γ and iNOS, and boosted the production of leishmania-specific IgG antibodies. Although no comparison was made with LAmpB treatment, it is clear that the encapsulation of ursolic acid offers a superior drug formulation with reduced toxicity and increased parasiticidal effect (Jesus et al., 2021).

Given the therapeutical benefits and the regulatory approval of LAmpB formulations, liposomes have been extensively employed for cell-targeting studies. De Oliveira et al. discovered that liposomes functionalized with hyaluronic acid (HA) enhanced drug accumulation by targeting infected cells. Activated/inflammatory macrophages overexpress the surface glycoprotein CD44 that specifically binds to HA. Biodistribution assays showed abundant hepatic and splenic HA-liposome accumulation after 24 h from i.v. injection of healthy and infected animals. HA-liposomes were also detected in the infected paws of BALB/c mice, which was a result of intense macrophage recruitment associated with the extensive inflammation in the infected tissue. when applied topically, HA-liposomes were able to cross the stratum corneum and reach the epidermis of healthy pig skin (de Oliveira et al., 2020).

Leishmania-specific drugs for topical application such as cream of paromomycin sulfate—Leshcutan—have shown a limited efficacy to treat cutaneous leishmaniasis. In fact, the stratum corneum is the main barrier to topical and transdermal drug delivery. Also, biomacromolecules, such as AmpB, were not able to cross the stratum corneum given their poor tissue permeability. Hypodermic injection of drugs is recommended in specific cases of CL, particularly for non-ulcerating wounds during acute infection (Minodier and Parola, 2007). The pain associated with injection, in addition to the side and toxic effects of the drug, reduces patient compliance. As an alternative, dissolvable carboxymethylcellulose/polyvinylpyrrolidone microneedles have been studied to deliver AmpB to the epidermis/dermis of CL lesions. Pyramidal microneedles measuring around 600 µm are able to pierce rat skin without bending or fracturing, and are almost entirely dissolved by the interstitial fluid after 30 min. Moreover, micropores created by microneedles were completely resealed after 30 min, without causing any detectable irritation reaction like erythema and swelling. The application of topical AmpB after pre-treating the skin using unloaded microneedles did not offer any quantitative advantage over drug delivered by loaded microneedles. By delivering around 87% of the AmpB contained in the microneedles, up to 3 daily administrations would be necessary to reach an intralesional AmpB concentration similar to that provided by standard AmpB treatment (Zare et al., 2021).

Chitosan (CHI) nanoparticles have also been evaluated for the treatment of leishmaniasis, especially after the FDA approved the use of wound dressings containing CHI. Although AmpB-loaded chitosan nanoparticles (AmpB-CHI) were shown to be safer and more effective than standard LAmpB treatment, neither AmpB-CHI nor LAmpB were able to penetrate the skin to deliver AmpB. The accumulation of phagocytes from inflammation in infected tissues may have contributed to the increased nanoparticle uptake observed in infected tissues after topical administration. On the contrary, intravenous administration of AmpB-CHI increased the intralesional concentration of AmpB and decreased parasite burden in comparison to LAmpB treatment (Riezk et al., 2020).

Topical treatment was also investigated by Kannan et al., who proposed an innovative *in vitro* method to evaluate the parasite-repellent power of Fe_2_O_3_ nanoparticles coated or not with polyethyleneimine (PEI) and incorporated in cream and gel formulations. In an agar plate assay, the authors observed that only coated nanoparticles were able to arrest the parasite migration by killing them. They also reported that PEI worked as a proton sponge in the lysosome compartment, increasing osmotic pressure and causing mitochondrial damage. Only trypanosomatids present a single lysosome-like structure, also known as a multivesicular tube in promastigotes and a megasome in amastigotes (Besteiro et al., 2007; Ueda-Nakamura et al., 2007). The rupture of this organelle releases hydrolytic enzymes that degrade the entire parasite in a few minutes. The active nano-based formulation, also called Nano-Leish-IL, showed a comparable antileishmania effect of Leishcutan in a murine model of acute leishmaniasis, as revealed by qPCR analysis. Dermoscopic imaging showed wound shrinkage after treatment with Nano-Leish-IL when compared to lesion volumes of untreated mice. In addition, Nano-Leish-IL was able to prevent the development of lesions when administered immediately after infection. As paromomycin-resistant Leishmania has been reported, the authors stated that the primary advantage of Nano-Leish-IL over the available paromomycin cream is the lack of evidence about resistance mechanisms induced by nano-drugs (Kannan et al., 2021). However, considering that the emergence of drug-resistant microorganisms typically occurs after an extended period of treatment, this finding must be interpreted with caution.

Although the topical administration of antileishmanial drugs is still controversial due to the limited ability to penetrate the deepest epithelial layers, the mechanism of action of the loaded drug can help to overcome typical permeability issues. Topical administration of CHI nanoparticles loaded with a NO donor promoted significant reduction in lesion thickness and parasite burden, without macroscopic signals of inflammation. CHI nanoparticles protected the encapsulated NO from degradation in addition to allowing controlled release for up to 21 days after only one administration. NO plays a central role in microbicidal macrophage activity, but increased microvascular permeability promoted by NO may have contributed to the success of NO-loaded CHI-nanoparticles (Cabral et al., 2021). Considering that parasites are not restricted to the lesion in chronic CL, additional studies should evaluate the potential of NO-donor CHI-nanoparticles in combination with systemic therapies. Increased NO production was also considered the primary mechanism for the successful topical administration of copper nanoparticles (CuNPs) in a murine model of cutaneous leishmaniasis. The reduction in parasite burden was potentialized by combining topical CuNPs and intralesional administration of free meglumine antimoniate (Glucantime), a first-line drug to treat cutaneous leishmaniasis (Albalawi et al., 2021).

CHI nanoparticles have also been developed as a drug carrier for the oral delivery of antileishmanial drugs. Sarwar et al. developed thiolated CHI (Thi-CHI) to enhance its mucoadhesion and permeation. Thiolated polymers also act by inhibiting the trypanothione-reductase system (TR) that prevents the accumulation of antimonial drugs. To target infected macrophages, Thi-CHI was also grafted with mannose residues since mannose receptors are increased in Leishmania-infected macrophages. The Glucantime (Glu)-loaded thiolated nanocarriers (Glu-Thi-CHI) improved penetration into the intestinal barrier in comparison to the same dose of oral Glu. The increased hepatic and serum concentration of stibogluconate after Glu-Thi-CHI treatment reduced the hepatic and splenic parasite burden more efficiently than the standard intraperitoneal treatment (Shoaib Sarwar et al., 2020). Considering the renal toxicity caused by Glu, Cosco et al. developed biocompatible Glu-loaded aqueous-core polylactic acid (PLA) nanocapsules in an attempt to reduce kidney toxicity. Glu-loaded PLA nanocapsules injected intraperitoneally in healthy mice readily accumulated in the reticuloendothelial system, showing a lower accumulation in kidneys resulting in a higher plasma half-life. When administered to *L infantum*-infected mice, Glu-loaded nanocapsules promoted significant reduction in parasite burden in the liver and kidneys when compared with standard Glu, but failed to improve the leishmanicidal activity in the spleen. However, by using lower a lower dose of Glu in the nanostructured formulations, the authors suggest that the Glu dosing could be revisited if delivered by a nano-based formulation, such as the aqueous-core PLA nanocapsules, in order to prevent the typical treatment toxicity (Cosco et al.).

Microparticles of β-1,3-glucan, an inexpensive raw material in *Saccharomyces cerevisiae* yeast cell walls, were tested as oral carriers for hydrophobic drugs against *L. infantum*-infected macrophages (Volpato et al., 2018; Scariot et al., 2019a). β-1,3-glucan is recognized by the dectin-1 receptor on the cell membrane of phagocytic mononuclear cells, promoting the internalization of those carriers by endocytosis. *L. infantum*-infected animals were treated with particles consisting of a bioactive thiophene encapsulated in glucan derived from yeast cell walls (YCWP). In the intestine, glucan from YCWP is recognized by dectin-1 receptors on microfold cells residing in Peyer’s patches. Microfold cells transferred the particles to lymphoid organs (e.g. bone marrow, spleen, lymph nodes), which are the organs targeted by Leishmania. The intense proinflammatory response triggered by the thiophene-loaded YCWP treatment in the spleen and liver promoted a significant reduction in parasite burden in both organs. No toxicity was reported in healthy animals (Scariot et al., 2019b).

Nanostructures may also exert antileishmanial activity in the absence of antileishmanial drugs by triggering an immunomodulatory response. Ramos et al. reported that spherical carbon nanostructures (fullerol) were able to control hepatic parasite growth by generating a pro-inflammatory response in a murine model of visceral leishmaniasis. To improve the delivery of fullerol in the liver and spleen, the nanostructure was encapsulated in unilamellar liposomes, intensifying the fullerol potential in reducing the hepatic parasite load. The detection of increased levels of IL-1β in the serum suggested that fullerol-loaded liposomes potentiated the macrophage activation, resulting in stronger activity of inducible nitric oxide synthase and parasiticidal effect (Ramos et al., 2021).

## American trypanosomiasis: Chagas disease

**Figure 2:**
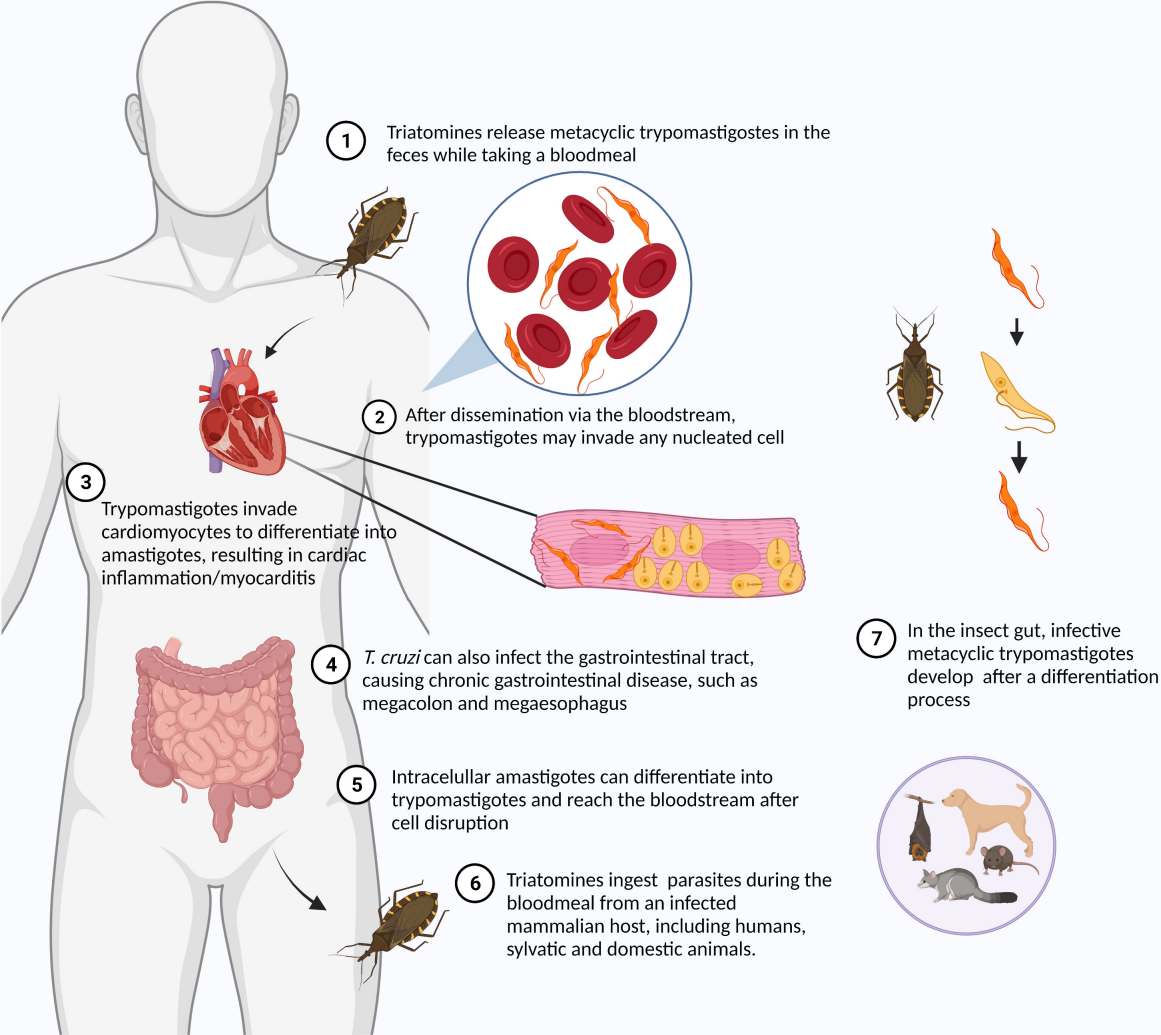
Life cycle of *Trypanosoma cruzi*. The life cycle of *T. cruzi* alternates between the insect vector – Triatomines or “kissing bugs” – and the mammalian host. Metacyclic trypomastigotes are found in insect feces released during the bloodmeal. The parasite reaches the bloodstream through the bite wound or conjunctiva. Once in the bloodstream, *T. cruzi* can invade any nucleated cells. After invasion, the parasite differentiates into amastigote and multiplies in the host cell cytoplasm. After some cycles of multiplication, intracellular parasites differentiate into trypomastigotes. Trypomastigotes are released in the bloodstream after the host cell disruption being ingested by triatomines while taking a bloodmeal from an infected mammalian host. In the insect gut, different developmental stages of parasites can be found, including epimastigotes and metacyclic trypomastigotes. Created with Biorender.com.

American trypanosomiasis or Chagas disease (CD) is a vector-borne disease caused by the protozoan *Trypanosoma cruzi*. Although originally endemic to the Americas, CD has spread to disparate regions of the globe through the migration of infected individuals, with 8 million people infected worldwide. Up to 12,000 infected people die annually of cardiac complications caused by the chronic *T. cruzi* infection (PAHO, 2022). It is estimated that about 300,000 people in the United States harbor this parasite, most having unknowingly acquired the infection in South and Central America with a small number *via* autochthonous infection. *T. cruzi* is also found in a large number of wild mammals throughout the Americas (Montgomery et al., 2016). *T. cruzi* is typically transmitted through contaminated feces of an insect vector called triatomines or “kissing bugs,” but oral transmission through ingestion of food contaminated with Triatomine feces, blood transfusions, and vertical transmission have also contributed. During the bloodmeal, male and female triatomines release feces containing flagellated parasites called trypomastigotes, which penetrate the body through the bite wound or through mucous membranes (Martín-Escolano et al., 2022). As an obligate intracellular parasite, *T. cruzi* invades cells at the inoculum site (e.g. macrophages, fibroblasts, epithelial cells), starting the acute infection. Within the host cell, parasites escape the parasitophorous vacuole to the cytoplasm, differentiate to nonflagellated amastigotes and divide by binary fission (**Figure 2**). Approximately 30% of infected individuals develop chronic symptoms of CD, including dilated cardiomyopathy, arrhythmias, thromboembolic events, and digestive disorders like megaesophagus and megacolon syndromes (Tanowitz et al., 2015; Pérez-Molina and Molina, 2018), resulting from decades of gradual organ injury, parasite persistence and parasite- and host-directed immunity and inflammation. Currently, there is no approved vaccine for *T. cruzi*. The autoimmunity associated with the development of chronic chagas cardiomyopathy (De Bona et al., 2018) has hindered the development of attenuated or killed parasite vaccines for *T. cruzi*. Several *T. cruzi* proteins are able to promote cardiac damage in the absence of live parasites by inducing autoimmunity in animal models (Cunha-Neto et al., 1996; Bonney et al., 2011). Fortunately, a number of other recombinant *T. cruzi* proteins as well as attenuated *T. cruzi* parasites are emerging as potentially safe strategies for the development of a CD vaccine (Sánchez-Valdéz et al., 2015; Rios et al., 2019; da Costa et al., 2021; Rodrigues da Cunha et al., 2021). Researchers are also testing mRNA vaccines after their successful use in COVID-19 (Diaz-Hernandez et al., 2022; Maldonado et al., 2022).

The early detection of CD is critical for treatment success. If administered during acute *T. cruzi* infection, antichagasic drugs are up to 80% effective in clearing the parasite. (Meymandi et al., 2018). Although acute *T. cruzi* infection can be easily detected by direct observation of the parasite in the peripheral blood, acute CD is rarely diagnosed due to the nonspecific symptoms and autoresolution in 4 to 8 weeks (Ribeiro et al., 2012). The diagnosis of infection during chronic CD is typically done by detection of *T. cruzi*-specific antibodies (Angheben et al., 2019). Stable, more convenient and less expensive diagnostic methods having high sensitivity and specificity, such as rapid antibody tests, would be extremely helpful for detection of CD. Nanotechnology has been applied to the development of innovative techniques to detect *T. cruzi* infection (Morilla and Romero, 2015). A highly-publicized nano-based test called Chunap (Chagas urine nanoparticle test) composed of hydrogel nano-porous particles functionalized with Trypan Blue sequesters and concentrates *T. cruzi* antigens in urine. Antigens enter the particles depending on their tertiary structure and molecular weight and the concentrated antigens are identified by western blotting, showing similar sensitivity to PCR. The particles also protect *T. cruzi* antigens in urine against enzymatic degradation. The non-invasive and convenience of urine collection is one of the advantages of Chunap over serology or PCR from blood, especially for infants (Castro-Sesquen et al., 2014; Castro-Sesquen et al., 2016).

During the chronic stage of CD, variable levels of anti-*T. cruzi* antibodies are present in the serum. To improve the sensitivity and specificity of diagnostic methods for chronic CD, indium phosphide (InP) nanowire biosensors were developed based on field-effect transistors. In this system, changes in conductivity can be detected after a biomarker, e.g. a *T. cruzi* antigen, binds to a specific bioreceptor placed on the nanoscale biosensor. InP nanowires were first prepared by surface biofunctionalization with ethanolamine and poly(ethyleneglycol) (PEG). Next, IBMP-8.1, a specific biomarker found in the serum of *T. cruzi*-infected humans, was covalently bound to the PEG-functionalized nanowires. Nanowires suspended in isopropanol were put over a metal electrode based on Ni/Ge/Au alloy. Nanowires were able to detect anti-IBMP-8.1 antibodies present in serum at concentrations as low as 6 fM, with excellent specificity. By comparison, standard diagnostic methods, such as ELISA and PCR, have sensitivity in the nanomolar range. The significant alteration in the electrical properties of the InP nanowire sensor after anti-IBMP-8.1 antibody attachment was the endpoint of this method (Janissen et al., 2017). More recently, Lima et al. conjugated gold nanoparticles to a silicon-based material (water-soluble charged silsesquioxanes) to be applied as nanotransducers in an electrochemical biosensor for the immunodetection of *T. cruzi* infection. The gold nanoparticles were covered by erythrocytes sensitized with *T. cruzi* antigens. When in contact with anti-*T. cruzi* antibodies in serum, the formation of an immunocomplex inhibits the redox reaction on the probe, resulting in lower electric current. Importantly, the developed biosensor did not show toxicity against red and white blood cells, and is therefore potentially safe for intravenous application. Regarding the applicability of this diagnostic tool in endemic areas of low-income countries, the electrochemical biosensor developed by Lima et al. could be easily adapted to portable devices. Additionally, because there is no need for complex sample preparation, the cost of this method should be lower than that estimated for most diagnostic approaches based on nanostructured materials (Lima et al., 2022).

Only two hydrophobic nitroimidazole prodrugs have been available to treat *T. cruzi*-infected patients since the 1970s — nifurtimox (NFX) and benznidazole (BNZ) (Bern, 2011). Although both drugs can effectively clear the acute *T. cruzi* infection, BNZ is the first-line treatment against chronic CD, being better tolerated than NFX. Nevertheless, the severe side effects, such as hepatic and renal damage, anorexia, immunosuppression, teratogenicity, neuropathy, dermatitis, etc., require cessation of treatment in up to 30% of patients (Coura and Castro, 2002; Castro et al., 2006; Ndayishimiye et al., 2021). BNZ toxicity results from the required administration of high oral doses of drug that are required to achieve a therapeutic plasma dose; this is largely due to the drug’s high hydrophobicity. Improvements in BNZ formulation have been achieved (Davanco et al., 2016; Bezerra et al., 2020; Amaral et al., 2021; Ndayishimiye et al., 2021) and micro- and nano-carriers have emerged as a strategy to decrease BNZ toxicity by overcoming poor aqueous solubility, increasing BNZ bioavailability and increasing concentration inside *T. cruzi* infected cells (Quezada et al., 2019; Seremeta et al., 2019; Pandian et al., 2021).

We found that BNZ- loaded poly(ethylene glycol)-block-poly(propylene sulfide) (PEG-b-PPS) polymersomes are effective at treating acute *T. cruzi* infection at BNZ doses 466-fold lower than with free BNZ. *In vitro* analysis revealed the cytoplasmic colocalization of PEG-b-PPS nanocarriers and parasites inside cardiomyocytes, consistent with previous reports that sulfide moieties of PPS can be oxidized by endolysosomal enzymes and migrate to the cytoplasm (Scott et al., 2012). In a murine CD model, standard BNZ and BNZ-loaded PEG-b-PPS polymersomes reduced *T. cruzi* infection; however, only the BNZ-loaded PEG-b-PPS polymersomes promoted a significant reduction of heart inflammation in comparison to untreated controls (Li et al., 2021). High daily doses of BNZ are required to reach effective plasma drug concentrations, which cause various types of toxicity. The low BNZ dose delivered by a weekly iv injection of BNZ-PEG-b-PPS polymersomes did not cause the weight loss and hepatotoxicity observed in mice treated with free BNZ. Similarly, microparticles of Eudragit, a copolymer derived from esters of acrylic and methacrylic acids, delivering 50 mg/kg/day of BNZ by the oral route, obtained by the spraying-drying method, were able to clear acute heart parasitosis, as well as decrease *T. cruzi*-specific antibody levels and heart inflammation. In contrast, treatment with free BNZ at the same dose only reduced parasitemia by 50%, with development of cardiac inflammation. BNZ Eudragit microparticles show a faster dissolution rate and BNZ release than the raw BNZ. The advantage of BNZ Eudragit microparticles over free BNZ may be due to the improvement in the pharmacokinetic properties, thus improving BNZ intestinal absorption, resulting in a more effective trypanocidal activity (Rial et al., 2021). The same research group also reported that 71% of *T. cruzi*-infected mice treated with oral poloxamer (P-188) nanoformulations of BNZ at a dose as low as 25 mg/kg/day did not show parasites in the blood based on PCR results; after immunosuppression, no parasitemia were detected in 40% of the animals when compared with the treatment using free BNZ at the same dose and route of administration. BNZ nanoformulation treatment was also able to reduce significantly the anti-*T. cruzi* antibody levels and the cardiac inflammation as well when compared to the same dose of free BNZ (Rial et al., 2017). Reduction in antibody production almost certainly results from suppressed parasitemia and therefore low antigen availability to stimulate *T. cruzi*-specific antibody production.

Likewise, increasing the drug solubility and intestinal absorption was the goal of Spósito et al. (2017), who developed self-emulsifying nanoformulations for oral drug delivery. The nanoformulations contained ravuconazole, a lipophilic drug possessing a short plasma half-life and strong trypanocidal activity. Self-emulsifying drug delivery systems are a mixture of oil, surfactant, and drug that form micelles of 100-250 nm under gentle agitation. Small droplets are formed in the gastrointestinal tract, improving dissolution, intestinal absorption, and bioavailability of the loaded drug. The potential toxicity of nanoemulsions containing ravuconazole was measured by the weight loss of infected and uninfected animals. Although Labrasol formulations were found to be toxic to healthy mice, their nanoemulsion containing an optimal concentration of Labrasol combined with ethanol as emulsifiers did not promote any weight loss (Spósito et al., 2017). Regarding infected animals, all treatments, including free ravuconazole, prevented the severe weight loss caused by the infection. Treatment efficacy was time-dependent: all drug treatments (free benznidazole, free ravuconazole, ravuconazole-loaded nanoemulsion) cleared the infection after 40 days. A short-term treatment (30 days) revealed the superiority of ravuconazole-loaded nanoemulsions over treatment with free ravuconazole. Although additional toxicological analysis could further show the benefits of self-emulsifying drug delivery systems, the Spósito et al. investigation indicates that a self-nanoemulsifying system can overcome many limitations intrinsic to orally-administered lipophilic drugs (Spósito et al., 2021).

Immunomodulators, such as imiquimod (IMQ), have also been investigated against acute CD. Parra et al. showed that subcutaneous treatment of infected mice with IMQ incorporated in nanoarchaeosomas (NCH), oligolamellar nanovesicles produced with lipids isolated from archaebacteria, induces a protective Th-1 response against acute *T. cruzi* infection, promoting a significantly lower mortality rate and parasitemia than subcutaneous free IMQ treatment or no treatment. As IMQ-NCH caused less muscle inflammation and fibrosis than oral free BNZ, the combination of IMQ with trypanocidal drugs may reduce parasitemia/parasitosis and also reduce tissue inflammation in chronic CD (Parra et al., 2020).

BNZ treatment is recommended for chronic CD by many clinicians, but it remains controversial due to limited ability of the treatment to eliminate parasites entirely, together with the potential for severe side effects of the drug. The BENEFIT (Benznidazole Evaluation for Interrupting Trypanosomiasis) trial concluded that treatment of chronic CD patients does not prevent the progression of cardiomyopathy (Bonney and Engman, 2015; Morillo et al., 2015; Santos et al., 2020a). However, a long-term follow-up study revealed that BNZ treatment was associated with a lower incidence of the cardiac form of CD in patients treated from the indeterminate stage, suggesting that BNZ treatment can prevent the development of cardiovascular events if administered in patients without electrocardiographic abnormalities (Hasslocher-Moreno et al., 2021). Chronic chagasic cardiomyopathy is the primary concern for chronic patients and, for that reason, effective treatment should achieve a decline of cardiac inflammation in addition to the reduction of parasitosis as endpoints. The potential anti-inflammatory activity of natural molecules, such as curcumin (CUR), has been explored for treatment of chronic chagasic myocarditis. Hernandez et al. found that CUR encapsulated in poly(lactic-co-glycolic acid) nanoparticles (PLGA-NPs) reduced chronic chagasic inflammation caused by *T. cruzi* and optimized the antiparasitic effect of suboptimal doses of BNZ after oral administration in infected mice. CUR-PLGA-NP monotherapy downregulated proinflammatory cytokine levels but failed to reduce cardiac parasitosis while free BNZ significantly reduced parasitemia without promoting an anti-inflammatory response. The co-administration of CUR-PLGA-NPs and free BNZ resulted in improved trypanocidal activity and a decrease in the pro-inflammatory response, reducing heart fibrosis and cardiac hypertrophy. These findings revealed that molecules that suppress the release of inflammatory mediators should be explored in combination with antiparasitic drugs to treat chagasic inflammation and chronic parasitosis simultaneously (Hernandez et al., 2021).

In another study using the poloxamer (P-188) nano-based platform, Rial et al. compared the effect of continuous and intermittent oral administration of reduced doses of free BNZ and BNZ P-188-NPs in chronically infected mice. Intermittent administration of BNZ P-188-NPs achieved a similar efficacy to free BNZ using a 15% lower BNZ dose. The authors hypothesized that the superior efficacy of a sustained release of lower doses administered intermittently was a consequence of a longer treatment period. This allowed for BNZ activity against the dormant amastigote stage of *T. cruzi* that dominates the chronic stage. These findings suggest that high BNZ doses and BNZ accumulation are not needed to have a trypanocidal effect, preventing the typical severe side effects of BNZ treatment (Rial et al., 2020). Similarly, García et al. found that daily doses of oral BNZ are not required to promote the trypanocidal effect in animals. The authors also developed a multiparticulate drug delivery system (MDDS) to improve the release of BNZ by oral administration. In general, the MDDS aims to control or modify the drug release based on coating drug particles or free drug in a matrix of one or more oppositely charged polymeric carriers, which by electrostatic interaction with the aqueous environment, creates soluble or insoluble complexes able to modulate their dissolution rate and release of drug. The incorporation of BNZ in Eudragit EPO-Eudragit L100, an MDDS employing two oppositely charged polyelectrolytes with different degradation rates, enhanced BNZ activity. This was given orally and achieved a sustained BNZ concentration, resulting in nearly undetectable cardiac parasite burden in mice at 150 dpi, even after immunosuppression. Additionally, by avoiding the high plasma concentration of BNZ and exacerbated oxidative stress in uninfected mice, the controlled release of BNZ helped reduce systemic inflammation and did not cause hepatotoxicity typically found in mice treated with free BNZ (Garcia et al., 2021).

Branquinho et al. developed and evaluated the efficacy of a natural lipophilic sesquiterpene lactone, Lychnopholide (LYC), encapsulated in biodegradable poly(ethylene glycol)-block-polylactic acid (PEG-b-PLA) and polycaprolactone (PCL) nanocapsules (NCs) against acute and chronic CD. PEG-b-PLA-PCL NCs increase the plasma concentration of LYC by preventing fast LYC degradation in plasma, avoiding the fast clearance after intravenous and oral administration. LYC-PEG-b-PLA-PCL NCs reduced parasitemia similarly to standard BNZ treatment, resulting in 75% and 87.5% of animals being cured during acute and chronic infections, respectively. Inflammatory processes and parasites were not detected in the hearts of chronically infected mice treated with LYC- PEG-b-PLA-PCL NCs at 12 mg/kg/day. Although biodistribution studies for LYC are not currently available, Branquinho et al. suggested that the enhanced permeability of LYC and LYC- PEG-b-PLA-PCL NCs biodistribution may have been influenced by the chagasic inflammation itself (Branquinho et al., 2020).

## Discussion and conclusions

Many studies have illustrated the benefits of nanotechnology to improve the delivery, toxicity, drug release, and biodistribution of leishmanicidal and trypanocidal drugs. As the spleen and liver are primary targets of VL, the accumulation of nanoparticles in these organs may be considered an advantage for leishmaniasis treatment. Not surprisingly, the employment of different animal models and methods to test leishmanicidal efficacy in terms of tissue-specific parasite burden does not allow direct comparison of treatments across studies. A good leishmaniasis prognosis is strictly dependent on a balanced helper T cell protective immune response. In contrast, damages caused by *Leishmania* infection in the vertebrate host are a consequence of an exacerbated Th-1 response while an exacerbated Th-2 response leads to a severe and untreatable infection. Immunomodulation promoted by engineered nanomaterials to restore the balance between Th-1 and Th-2 macrophage response has not been explored and thus affords an opportunity for future study. Similarly, few studies have investigated the potential and promising application of nano-based drug delivery systems to target *T. cruzi* or *Leishmania* antigens as well as infected cells specifically. This limitation may be associated with the plasticity in immune evasion typical of both parasites by downregulating antigen expression on the surface of infected cells, hindering the recognition of specific markers to be targeted by drug nanocarriers.

A large number of nano-based formulations have been employed to enhance solubility, improve drug biodistribution, and optimize the dose regimens of standard parasiticidal drugs. A similar approach was previously applied for the development of liposomal AmpB, used for decades to treat systemic fungal infections and leishmaniasis. However, liposomal AmpB systems have limited efficacy against intracellular *T. cruzi* (Clemons et al., 2017; Cencig et al., 2011; Morilla and Romero, 2015; Pund and Joshi, 2017; Quijia Quezada et al., 2019) partially because liposomes and *T. cruzi* do not reside in the same cellular compartment; *T. cruzi* amastigotes reside in the cytoplasm, while liposomes release AmpB inside the phagolysosome, where Leishmania replicates. Additionally, the high hydrophobicity of AmpB impairs its diffusion from the phagosome to the cytoplasm (Romero and Morilla, 2010). The emergence of BNZ-resistant *T. cruzi* strains and the high toxicity of available trypanocidal drugs justify the need for drug research and development in the treatment of both acute and chronic *T. cruzi* infection. Considering the heterogeneity of BNZ susceptibility observed among different *T. cruzi* strains, the use of different strains and animal models limits direct comparison of drugs and/or drug delivery systems. Some studies fail to show the benefits of nano-based formulations by ignoring the importance of adding an animal group treated with the free drug at the same dose proposed in the nanoformulation. Also, as recommended by the FDA, biodistribution and pharmacokinetic studies should be conducted in infected animals considering the significant physiologic differences between healthy animals and infected animals in both acute and chronic stages. For example, BNZ has a longer elimination half-life in chronically infected humans, suggesting that a lower BNZ dose may improve treatment tolerability without compromising the antiparasitic effect (Soy et al., 2015). Not surprisingly, the BENDITA clinical trial corroborates this approach (Torrico et al., 2021). Moreover, the evaluation of anti-inflammatory responses in chronic myocarditis and exploring sustained drug release from nanomaterials can support therapeutic development against chagasic cardiomyopathy. Unlike BNZ, NFX has not been explored using nanotechnology (Arrua et al., 2019). However, new studies are expected shortly since NFX was recently approved by the FDA for the treatment of CD in pediatric patients. Although the BERENICE consortium (Benznidazole and triazole research group for nanomedicine and innovation on CD) had developed and studied different nano-based drug delivery systems for six years providing useful information regarding BNZ properties, no tested formulation was successfully applied in preclinical studies (CORDIS, 2022). However, the interdisciplinary nature of nanotechnology research has made this field extremely versatile and innovative. As new knowledge and technology are being absorbed by this science, advanced engineered nanoparticle systems have been developed to induce a wide range of physiological and immunological responses.

However, regulatory challenges and the high price of innovative drug delivery systems are critical barriers for their implementation in the medical field (Kirtane et al., 2021). The market price of nano-based formulations is still higher than those of conventional medicines. The research and development of nanocarriers, as well as the technology applied on the pharmaceutical manufacturing of this products, is still considered expensive, discouraging translational research and clinical trials focused on diseases that affect low- and middle-income countries (Kumar et al., 2020). However, economic studies have revealed that a deeper cost-benefit analysis should be performed. One example is the use of LAmpB as a last-choice treatment for leishmaniasis in Brazil, where the National Public Health System database centralizes all information regarding direct costs of all available treatments, such as hospitalization period, medicine consumption, lab and imaging tests, medical appointments, and professional fees. Those studies show that LAmpB treatment significantly minimizes indirect costs not only by reducing the side effects and the loss of economic productivity, but also by increasing the cure rate, preventing relapsed leishmaniasis and future hospitalizations. The cost-effectiveness analysis is clear about the economic and social advantages of using LAmpB as the first-line treatment against leishmaniasis in that country (Mistro et al., 2016; Mistro et al., 2017; de Carvalho et al., 2020). Thus, additional cost-effectiveness studies are needed to clarify the benefits of nanotechnological medical advances in terms of budget for different health conditions, including other NTDs.

In summary, the preliminary success of nano-based drug delivery systems discussed here is expected to stimulate translational research using innovative engineered nanomaterials to treat leishmaniasis, acute and chronic CD. However, breaking the paradigm of “high-priced technology” is crucial to encourage investments in translational research and clinical trials. Considering the progress made in the last few decades in terms of academic research and development of nanomaterials against Trypanosomatid infections, the potential of nanotechnology against NTDs is high.

## Author contributions

DS searched the literature and wrote the initial draft of the manuscript. AS, JZ, XL, ES and DE edited the manuscript. All authors contributed to the article and approved the submitted version.

## Funding

This work was supported in part by US Public Health Service Grant R21-AI144529 (ES, DE), a Catalyst Award from the Northwestern University McCormick School of Engineering (ES).

## Conflict of interest

The authors declare that the research was conducted in the absence of any commercial or financial relationship that could be construed as a potential conflict of interest.

## Publisher’s note

All claims expressed in this article are solely those of the authors and do not necessarily represent those of their affiliated organizations, or those of the publisher, the editors and the reviewers. Any product that may be evaluated in this article, or claim that may be made by its manufacturer, is not guaranteed or endorsed by the publisher.
